# Three-Dimensional High-Resolution Laser Lithography of CsPbBr_3_ Quantum Dots in Photoresist with Sub-100 nm Feature Size

**DOI:** 10.3390/nano15070531

**Published:** 2025-03-31

**Authors:** Boyuan Cai, Haoran Jiang, Run Bai, Shengting Zhu, Yinan Zhang, Haoyi Yu, Min Gu, Qiming Zhang

**Affiliations:** 1 School of Artificial Intelligence Science and Technology, University of Shanghai for Science and Technology, Shanghai 200093, China; 222180338@st.usst.edu.cn (H.J.); 232200331@st.usst.edu.cn (R.B.); 212180316@st.usst.edu.cn (S.Z.); zhangyinan@usst.edu.cn (Y.Z.); gumin@usst.edu.cn (M.G.); 2 Institute of Photonic Chips, University of Shanghai for Science and Technology, Shanghai 200093, China

**Keywords:** perovskite luminescent quantum dots, high-resolution three-dimensional patterning, femtosecond laser direct writing, multi-level fluorescence modulation

## Abstract

Perovskite quantum dots (PQDs), with their excellent optical properties, have become a leading semiconductor material in the field of optoelectronics. However, to date, it has been a challenge to achieve the three-dimensional high-resolution patterning of perovskite quantum dots. In this paper, an in situ femtosecond laser-direct-writing technology was demonstrated for three-dimensional high-resolution patterned CsPbBr_3_ PQDs using a two-photon photoresist nanocomposite doped with the CsPbBr_3_ perovskite precursor. By adjusting the laser processing parameters, the minimum line width of the PQDs material was confirmed to be 98.6 nm, achieving a sub-100 nm PQDs nanowire for the first time. In addition, the fluorescence intensity of the laser-processed PQDs can be regulated by the laser power. Our findings provide a new technology for fabricating high-resolution display devices based on laser-direct-writing CsPbBr_3_ PQDs materials.

## 1. Introduction

In recent years, metal halide perovskite (MHP) materials and perovskite quantum dots (PQDs) have become a new generation of materials for manufacturing high-performance photonic and optoelectronic devices due to their excellent optoelectric properties, including their tunable bandgap, large light absorption coefficient, long carrier duration, and high photoluminescence efficiency [1,2,3,4]. In particular, solution-processed MHP QDs, especially when prepared by the colloidal chemistry route, have shown great potential beyond traditional QDs in many cutting-edge technologies, such as display, light-emitting diodes, photovoltaics, anti-counterfeiting and lasers [5,6,7]. However, the patterning of MHP for the miniaturization and integration of nanophotonic applications is mainly focused on two-dimensional top-down fabrication technologies such as photolithography, inkjet printing, and nanoimprinting lithography. Specifically, photolithography usually requires sophisticated steps (exposure and development) and lasts for a long time period, which may lead to the deterioration of MHP. Moreover, the resolution is limited to the micrometer scale [8]. The research team fabricated latent patterns with visible-light transparency and near-infrared (NIR) responsiveness by the direct inkjet deposition of indium tin oxide (ITO) nanoparticle ink onto transparent polyethylene naphthalate (PEN) substrates. However, the printing resolution remained limited to 75 μm [9], dictated by ink viscosity constraints and nozzle resolution limits. Nanosphere lithography (NSL) enables submicron resolution through optimized microsphere dimensions and etching protocols. However, its inherent template-dependent characteristics and fixed structural periodicity fundamentally limit its applicability in three-dimensional high-precision fabrication [10]. The comparison of our work with existing fabrication techniques is presented in Table S1 (Supplementary Materials). Thus, it is critical to develop a three-dimensional fabrication technique to precisely pattern perovskite with high-resolution feature sizes, which can hold promise for three-dimensional display and information storage [11,12,13].

To date, an ultrafast laser has been demonstrated to be an effective tool for direct spatiotemporal micromachining perovskite nanocrystals in transparent materials [14,15]. As a non-contact technology, the nonlinear absorption process induced by femtosecond laser pulses in a polymer or glass matrix can increase photothermal energy in local areas [16], thereby leading to the migration of perovskite ions and local crystallization of perovskite nanocrystals in the laser focus regions, which provides opportunities for three-dimensional patterning technology [17,18]. Recently, Liang Liu et al. reported a femtosecond laser-direct-writing technique (FsLDW) [19], which involves efficient, contactless, maskless, and depth-resolved micro-imaging [20,21]. Moreover, laser-direct-writing can flexibly fabricate arbitrary micro- and nanostructures on the same substrate. By in situ fabricating high-resolution patterned PQDs, a minimum line width of 1.58 μm can be achieved, providing an alternative option for the high-resolution imaging technology of PQDs [22]. Furthermore, recently, Hu and his colleagues managed to in situ fabricate a full-color perovskite quantum dot (PQD) array within a polyacrylonitrile (PAN) matrix by employing the femtosecond laser-direct-writing (FsLDW) technique [23]. Through this, they accomplished the high-resolution patterning of full colors with sub-diffraction characteristic sizes and even achieved a minimum size of 410 nm for this pattern, thus taking another step closer to a high-resolution patterned structure. However, there are still certain difficulties in achieving pixel sizes with a precision of hundreds of nanometers. The laser writing of perovskite QDs in the glass matrix usually requires additional heat annealing to produce luminescent perovskite nanocrystals. Even though the perovskite QDs can be generated via the one-step direct-laser-writing process in polymers, such as the PMMA or PVDF film, the resolution of the obtained perovskite QDs lines is usually limited in the sub-micrometer region due to the low diffusion of perovskite precursors in the polymer matrix [24]. To fulfill the application demands such as on-chip integration and ultra-high-resolution displays, it is highly desirable to develop new photocurable perovskite materials for laser lithography in a nanoscale feature size.

In this paper, we developed a laser-direct-writing strategy for three-dimensional high-resolution patterned PQDs based on CsPbBr_3_ perovskite precursors doped with two-photon photoresist [25]. By combining the two-photon polymerization effect of the photoresist and the femtosecond laser-induced crystallization of CsPbBr_3_ perovskite precursors, the minimum linewidth of the nanowire with CsPbBr_3_ PQDs can reach 98.6 nm with bright photoluminescent around the wavelength of 508 nm (full width at half maximum ~25 nm). The formation of laser-induced CsPbBr_3_ PQDs can also be confirmed by the Raman scattering spectrum and the PL spectrum. Furthermore, we investigated the impacts of laser power on the photoluminescent intensity of the CsPbBr_3_ PQD photoresist nanocomposite [26,27]. As the laser power increased, the fluorescence intensity of CsPbBr_3_ PQDs progressively intensified in the visible light range. Finally, a complicated fluorescence image of the USST school badge pattern was fabricated by laser writing, which can be applied to the fields of information storage, nonlinear optical neural networks, etc. Our results offer a promising solution for fabricating next-generation perovskite quantum dots with high resolution, providing significant potential for applications in optical data storage, 3D displays, and nonlinear optical neural networks [28].

## 2. Materials and Methods

**Figure 1:**
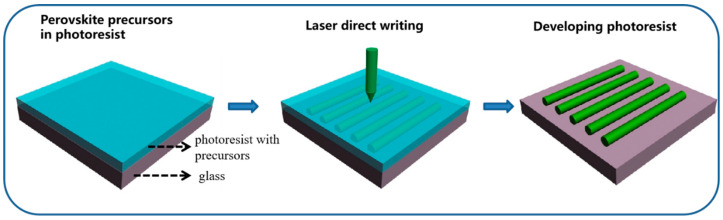
Schematic of the fs-laser writing of the doped CsPbBr_3_ perovskite precursor photoresist nanocomposites.

The perovskite photoresist nanocomposite prepared in this paper involves blending the precursor of perovskite CsPbBr_3_ into a two-photon photoresist material. The photoresist monomers have 50:50 wt% blend of triacrylate monomers (Sartomer SR9008 and SR368, Arkema Sartomer, Guangzhou, China); the photoinitiator is 4,4′-bis(di-n-butylamino)biphenyl (DABP) or E,E-1,4-bis[4-(di-n-butylamino)styryl]-2,5-dimethoxybenzene (DABSB) [29]. Then, the CsPbBr_3_ precursor solution is prepared using CsBr, PbBr_2_, and DMSO, and it is blended with the photoresist at the molar ratio of 1:4 to form a two-photon photoresist nanocomposite doped with the CsPbBr_3_ precursor. Figure 1 shows the schematic diagram of the fabrication process of the patterned CsPbBr_3_ PQDs with a focused femtosecond laser beam. The parameters of the laser used in this paper are a femtosecond pulsed laser beam with a wavelength of 517 nm (a repetition frequency of 2 MHz and a pulse width of 350 fs). The whole fabrication process includes three steps: first, the prepared precursor nanocomposite ink was dropped on a glass substrate; then, a 517 nm fs-laser with a 1.45 NA oil objective lens was applied for direct patterning with the perovskite photoresist nanocomposite to form CsPbBr_3_ PQDs nanostructures; and lastly, the residual precursor solution after the laser was processed was cleaned out with IPA and acetone solution, leaving the patterned photoresist with CsPbBr_3_ PQDs attached to the glass substrates. During the fabrication process, the laser can induce both the polymerization of the photoresist and the crystallization of the precursor of perovskite CsPbBr_3_. The perovskite photoresist nanocomposite samples after laser writing were characterized using the Raman scattering spectrum.

## 3. Results and Discussions

### 3.1. Optimization of the Minimum Line Width

**Figure 2:**
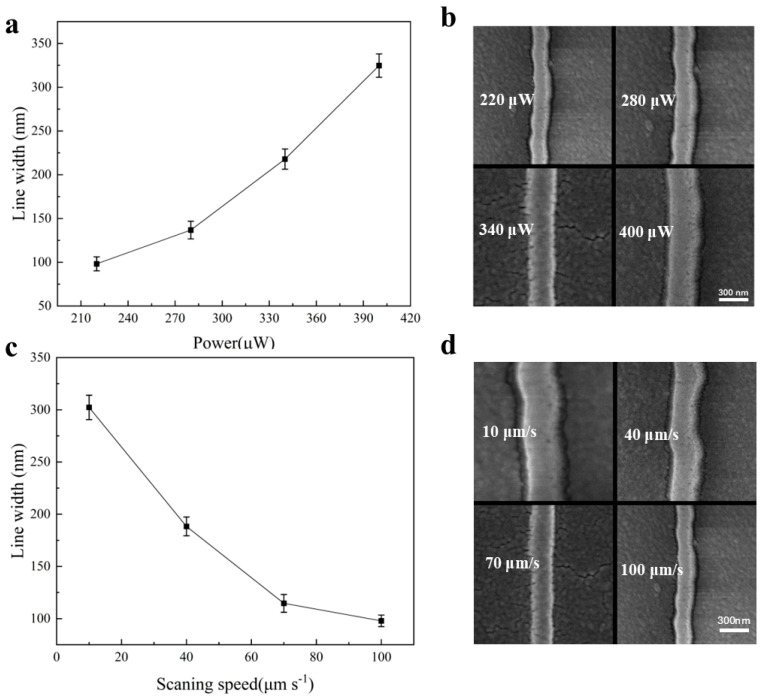
(**a**) Linewidth with different levels of laser-writing power. (**b**) SEM images of the nanowires doped with CsPbBr_3_ PQDs. (**c**) Linewidth of the nanowires with different laser scanning speeds. (**d**) SEM images of the nanowires doped with CsPbBr_3_ PQDs and different scanning speeds.

First of all, we confirmed the feasibility of the in situ formation of CsPbBr_3_ PQDs induced by the laser and optimized by laser fabrication parameters. The laser focus position should be controlled by the computer to adjust the fs-laser focus just on the perovskite photoresist nanocomposite liquid–glass interface at the beginning of the process of laser writing. Then, to investigate the high-resolution laser fabrication ability of the CsPbBr_3_ PQDs pattern, we analyzed the influence of laser power on the direct-laser-processed CsPbBr_3_ PQD nanowires. As shown in Figure 2a, with the scanning speed fixed at 100 μm/s, it can be found that the achieved line width of the PQD nanowires widened with the increase in the laser power. As the laser power increased from 220 μW to 400 μW, the line width of the obtained nanowire gradually increased from 98.6 nm to 328.2 nm. When the laser power was lower than 220 μW, the fabricated nanowire could be discontinuous and disappear after removing the residual precursor solution, which was due to the fact that the required laser energy could not reach the threshold of the two-photon polymerization of the photoresist. High laser power could lead to heat diffusion with explosive splashed fragments attaching to the focal area, affecting the integrity and clarity of the nanowires. Figure 2b shows the SEM images of the line width after the use of laser-direct-writing nanowires. Meanwhile, it was also proved that within the photoresist nanocomposite doped with the CsPbBr_3_ perovskite precursor, it still possessed the properties of two-photon polymerization [30].

Apart from the laser power, we also investigated the impact of scanning speed on the linewidth of the direct-laser-processed CsPbBr_3_ PQD nanowires. The scanning speeds were set as 10 μm/s, 40 μm/s, 70 μm/s, and 100 μm/s, respectively. The laser power was set as 220 μW according to the previous experimental results. As shown in Figure 2c, as the scanning speed decreased from 100 μm/s to 10 μm/s, the line width of the nanowires increased from 98.6 nm to 332.6 nm. Figure 2d shows the SEM images of the line widths of the nanowires after laser-direct writing. The average linewidths of the laser-fabricated nanowires were 302 nm, 188 nm, 114 nm, and 98.6 nm, corresponding to 10 μm/s, 40 μm/s, 70 μm/s, and 100 μm/s. And when the scanning speed was over 100 μm/s, the laser-fabricated nanowire could not be obtained successfully due to the insufficient laser exposure time. Through the optimization of the laser power and the scanning speed, a minimum linewidth of 98 nm of the CsPbBr_3_ PQD nanowires could be achieved using direct-laser-writing technology. Regarding the power lower than 220 μW and scanning speed faster than 100 μm/s, the continuous nanowire could not be obtained because the threshold of the two-photon polymerization for the photoresist was not reached.

### 3.2. Characterization of the CsPbBr_3_ Quantum Dots

**Figure 3:**
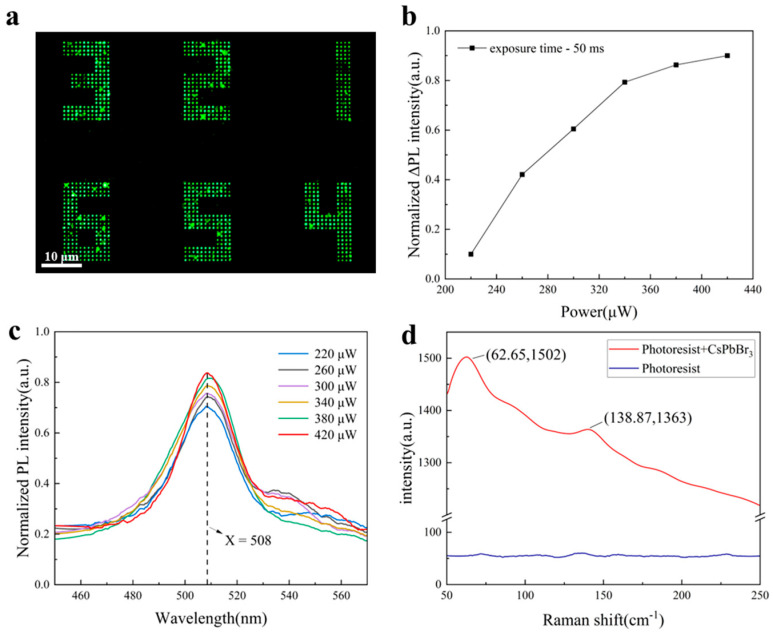
Optical characterization of perovskite: (**a**) Patterned dot array fluorescence image after laser writing; (**b**) normalized delta-photoluminescent intensity of PQDs with different laser-writing capabilities calculated from (**c**). (**c**) PL intensity of the PQDs at different laser powers. (**d**) In situ Raman scattering spectra of the pure photoresist versus laser-written PQD sample.

To demonstrate the formation of CsPbBr_3_ luminescent PQDs during laser-direct writing, the in situ PL spectrum of the PQDs generated by laser writing was measured systematically to investigate the patterning mechanism. The prepared perovskite photoresist nanocomposite material was highly transparent in the visible wavelength range, which indicates that the PQDs formation mechanism is highly related to nonlinear multiphoton absorption via the interaction with the fs-laser with high peak power. Here, an fs-laser of 517 nm with a pulse duration of 350 fs and repetition rate of 2 MHz was utilized to form 3D structures of the photoresist and the CsPbBr_3_ luminescent PQDs. The temperature rise in the focal area through nonlinear multiphoton absorption can be sufficient for the formation of PQDs during the photoresist patterning process. Figure 3a illustrates patterned PQD arrays with different laser powers. As shown in Figure 3b,c, the PQD nanodots with different levels of laser power (from 220 μW to 420 μW with an increased step of 40 μW) demonstrated a nonlinear-enhanced PL intensity. The fluorescence emission of PQDs is due to the recombination of electron–hole pairs in local electronic states caused by internal inhomogeneity-like defects or lattice distortions [31]. The PL intensity increased quickly with the laser power increasing from 220 μW to 340 μW, while the intensity increased slowly when the laser power increased to 420 μW. This may be due to the fact that more PQDs could be generated with the laser power increasing in the beginning period and then approaching the saturation status when the laser power further increased. We can also note that a lower laser power (<220 μW) cannot generate CsPbBr_3_ luminescent PQDs. In Figure 3c, similar PL spectra with different peak intensities can be observed at almost the same peak position of around 508 nm (full width at half maximum ~ 25 nm), consistent with the PL emission peak (approximately 510 nm) of CsPbBr_3_ PQDs, as reported by Yang Liu et al. [32]. Furthermore, Silvia G. Motti et al. [33] systematically demonstrated that reducing the size of CsPbBr_3_ nanocrystals induces a blue shift in the PL peak of the QDs due to quantum confinement effects. Notably, our material exhibits a 22 nm blue shift in the PL peak position compared to the bulk CsPbBr_3_ thin films (530 nm). This phenomenon aligns with the quantum confinement effects of CsPbBr_3_ PQDs, thereby validating the successful formation of the CsPbBr_3_ PQDs. The absorption spectrum of CsPbBr_3_ PQDs is shown in Figure S2 (Supplementary Materials). To further confirm the formation of CsPbBr_3_ PQDs, Raman scattering spectroscopy characterization was performed for the laser-writing PQDs. As shown in Figure 3d, CsPbBr_3_ Raman peaks at 62 cm^−1^ and 138 cm^−1^ were clearly obtained in the area with laser irradiation. Critically, no apparent Raman peaks were detected at these positions in undoped photoresist substrates, proving that our prepared nanocomposite can generate CsPbBr_3_ PQDs by laser processing. These two Raman scattering peaks can be ascribed to the vibration modes of [PbBr_6_]^4−^ octahedron and Cs^+^ cation movements. EDS characterization was conducted to verify the formation of CsPbBr_3_ PQDs (Figure S1, Supplementary Materials). It can be seen that the PQD array pattern illustrates a bright green fluorescence image under UV-365 nm light in Figure 3a, indicating its potential application in display, data storage, and optical encryption.

### 3.3. Fluorescence Display of CsPbBr_3_ PQD Photoresist Nanocomposite

**Figure 4:**
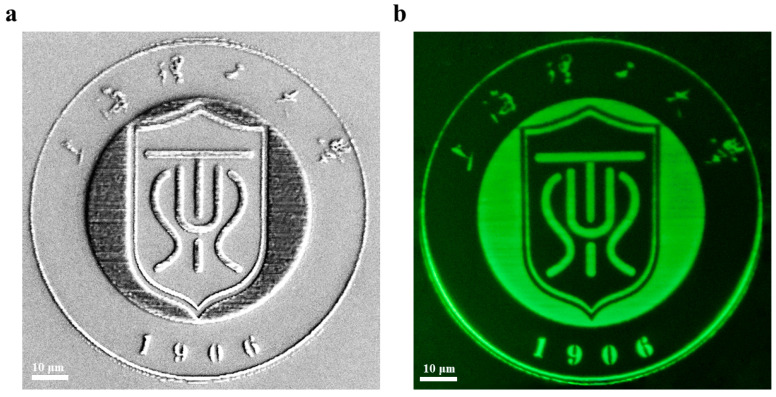
The patterned USST school badge after direct-laser writing: (**a**) SEM image. (**b**) Patterned fluorescence image under UV-365 nm light. Scale bar: 10 µm.

As previously discussed, the CsPbBr_3_ quantum dot-doped photoresist nanocomposite exhibits exceptional luminescent properties and laser writing is a simple and feasible technology for fabricating complicated micro/nano-patterns. Here, we also explore the potential application of the composite material in laser-direct writing for constructing more complicated patterns [34]. Using a laser power of 300 μW and a writing speed of 100 μm/s, a USST school badge pattern with a diameter of 100 μm was successfully fabricated. The SEM image of the school badge pattern, shown in Figure 4a, reveals a clear and well-defined structural morphology. Figure 4b presents the green fluorescence image of the pattern under 365 nm of UV light, displaying bright and uniform fluorescence, which confirms that the CsPbBr_3_ PQDs retained excellent luminescence performance during the laser-direct-writing process. Time-dependent photoluminescence intensity measurements of CsPbBr_3_ PQDs were performed, with detailed data shown in Figure S3 (Supplementary Materials). These results further validate the outstanding stability and fluorescence response capabilities of our perovskite precursor-doped nanocomposite, providing new opportunities for the development of high-performance photoresist materials. Additionally, the laser-fabricated pattern is not clear under sunlight, but it can illustrate bright green fluorescence under UV-365 nm light, implying its potential applications in data storage and optical encryption.

## 4. Conclusions

In conclusion, the applicability of in situ direct fs-laser writing technology for the 3D high-resolution patterning of CsPbBr_3_ PQD photoresist nanostructures has been demonstrated using the 517 nm femtosecond laser. The CsPbBr_3_ PQD photoresist nanowire with a minimum linewidth of 98.6 nm can be achieved with a laser power of 220 μW and a scanning speed of 100 μm/s. The PL intensity in the laser-constructed regions can be adjusted by the laser writing power, enabling the fabrication of fine patterns through laser processing. The direct laser writing technology combined with our perovskite photoresist nanocomposite demonstrated here may pave the way for the application of CsPbBr_3_ QD photonic devices in high-capacity optical data storage, 3D displays, and nonlinear optical neural networks.

## Data Availability

All the data supporting the findings of this study are presented in this article.

## Supplementary Materials

The following supporting information can be downloaded at: https://www.mdpi.com/article/10.3390/nano15070531/s1, Figure S1. EDS Spectrum of CsPbBr_3_ PQDs Sample. Figure S2. The absorption spectra of CsPbBr_3_ PQDs before and after laser irradiation. Figure S3. Temporal evolution of fluorescence intensity in CsPbBr_3_ PQDs monitored over 20 days post-fabrication. Table S1. Comparison of our work with data from existing fabrication techniques. References [35,36] are cited in the supplementary materials.
