# Supplementary material for: Three-Dimensional High-Resolution Laser Lithography of CsPbBr3 Quantum Dots in Photoresist with Sub-100 nm Feature Size
Source: Nanomaterials (Basel). 2025 Mar 31;15(7):531. doi: 10.3390/nano15070531 (PMC11990146; doi:10.3390/nano15070531)
Supplement: Supplementary file 1 [file nanomaterials-15-00531-s001.zip › nanomaterials-3515045-supplementary.pdf]

Supplementary Materials

Three dimensional high-resolution laser lithography of $CsPbBr_3$ quantum dots in photoresist with sub-100 nm feature size

Boyuan Cai [1,2,*,†], Haoran Jiang [1,2,†], Run Bai [1,2], Shengting Zhu [1,2], Yinan Zhang [1,2], Haoyi Yu [1,2,*], Min Gu [1,2], and Qiming Zhang [1,2,*]

[1] School of Artificial Intelligence Science and Technology, University of Shanghai for Science and Technology, Shanghai, 200093, China
[2] Institute of Photonic Chips, University of Shanghai for Science and Technology, Shanghai, 200093, China
* Corresponding authors Email: caiboyuan@usst.edu.cn; Haoyiyu@usst.edu.cn; qimingzhang@usst.edu.cn;
† These authors contributed equally to this work.

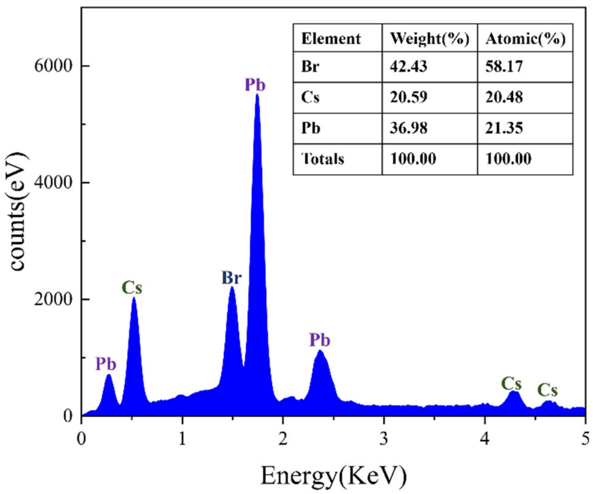

Figure S1. EDS Spectrum of $CsPbBr_3$ PQDs Sample.

The EDS characterization of $CsPbBr_3$ PQDs has been shown in Figure S1. The inset in Figure S1 illustrates the measured weight ratios (Cs:Pb:Br ≈ 21%:37%:42%) and molar ratios derived from semi-quantitative EDS analysis. These experimental weight percentages show excellent agreement with the theoretical values (23%:36%:41%) calculated for $CsPbBr_3$ materials. The atomic ratio of Cs: Pb: Br is 1:1:2.8, which is very close to the stoichiometric ratio of 1:1:3 in the $CsPbBr_3$ crystal structure. The distinct characteristic peaks corresponding to all three constituent elements can be clearly observed in the EDS spectrum, confirming the successful formation of $CsPbBr_3$ PQDs with proper elemental composition.

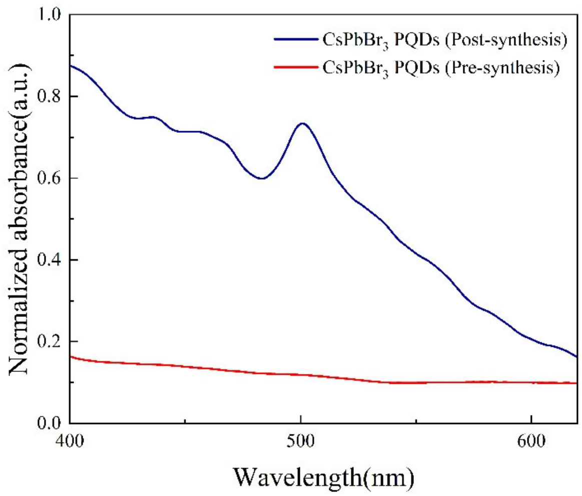

Figure S2. The absorption spectra of $CsPbBr_3$ PQDs before and after laser irradiation.

Figure S2 presents the absorption spectra of $CsPbBr_3$ PQDs before and after formation. The crystallized perovskite material exhibits a distinct absorption peak at approximately 500 nm, consistent with the intrinsic absorption characteristics of $CsPbBr_3$ PQDs. In contrast, the precursor mixture prior to laser irradiation shows a featureless absorption spectrum without distinct peaks, confirming the laser-induced generation mechanism of QDs. Notably, the absorption peak demonstrates an 8 nm blue shift compared to the photoluminescence (PL) emission peak at 508 nm, characteristic of the Stokes shift phenomenon in semiconductor materials. The observed Stokes shift arises from excited-state energy losses caused by non-radiative relaxation processes, such as electron-phonon interactions.

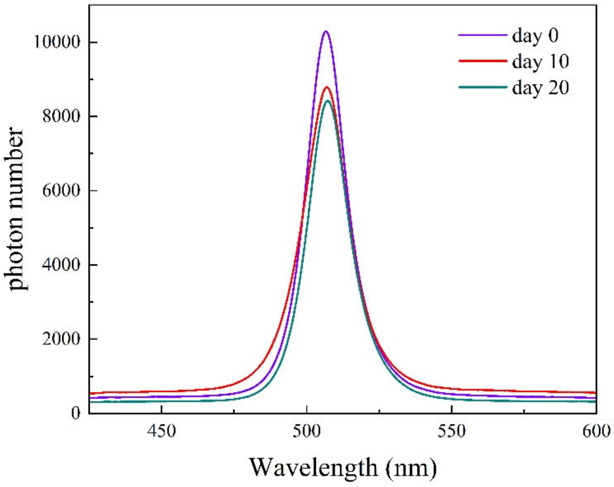

Figure S3. Temporal evolution of fluorescence intensity in $CsPbBr_3$ PQDs monitored over 20 days post-fabrication.

The time-dependent fluorescence spectrum of $CsPbBr_3$ PQDs is illustrated in Figure S3.

Our systematic 20-day monitoring of fluorescence intensity, as presented in Figure S3, revealed a decay of approximately 15.5% at 10 days and 18.6% at 20 days. This degradation demonstrates the material's long-term operational stability rather than rapid short-term deterioration.

The following table S1 compares our work with other existing fabrication techniques, demonstrating the high-resolution processing precision of laser direct writing.

Table S1. Comparison of our work with data from existing fabrication techniques.

Material	Method	Minimum Resolution	Ref.
$CsPbBr_3$	Direct photolithograph	1 μm	1
In_2O_3	Inkjet printing	2 μm	2
$MAPbI_3$	Nanosphere lithography	250 nm	3
PET films	EHD inkjet-printing	50 μm	4
$CsPbBr_3$	Laser direct writing	1.58 μm	5
$CsPbI_3$ in PMMA	Laser direct writing	900 nm	6
$CsPbBr_3$	Laser direct writing	100 nm	Our work

1. Pan J A, Ondry J C, Talapin D V. Direct optical lithography of CsPbX3 nanocrystals via photoinduced ligand cleavage with postpatterning chemical modification and electronic coupling[J]. Nano Letters, 2021, 21(18): 7609-7616.
2. Kim S Y, Kim K, Hwang Y H, et al. High-resolution electrohydrodynamic inkjet printing of stretchable metal oxide semiconductor transistors with high performance[J]. Nanoscale, 2016, 8(39): 17113-17121.
3. Yao Z, **ong Y, Kang H, et al. Tunable Periodic Nanopillar Array for MAPbI3 Perovskite Photodetectors with Improved Light Absorption[J]. ACS omega, 2024, 9(2): 2606-2614.
4. Khan A, Rahman K, Ali S, et al. Fabrication of circuits by multi-nozzle electrohydrodynamic inkjet printing for soft wearable electronics[J]. Journal of Materials Research, 2021: 1-11.
5. Liang S Y, Liu Y F, Wang S Y, et al. High-resolution in situ patterning of perovskite quantum dots via femtosecond laser direct writing[J]. Nanoscale, 2022, 14(4): 1174-1178.
6. Zhan W, Meng L, Shao C, et al. In situ patterning perovskite quantum dots by direct laser writing fabrication[J]. Acs Photonics, 2021, 8(3): 765-770.
